# Metabolic Syndrome and Hypertension Resulting from Fructose Enriched Diet in Wistar Rats

**DOI:** 10.1155/2017/2494067

**Published:** 2017-04-11

**Authors:** Julie Dupas, Annie Feray, Christelle Goanvec, Anthony Guernec, Nolwenn Samson, Pauline Bougaran, François Guerrero, Jacques Mansourati

**Affiliations:** ^1^EA 4324-Optimisation des Régulations Physiologiques, Institut Brestois Santé Agro Matières, Université de Bretagne Occidentale, 6 avenue Victor Le Gorgeu, 29238 Brest Cedex 3, France; ^2^UFR Sciences du Sport et de l'Education, 20 avenue Victor Le Gorgeu, 29238 Brest Cedex 3, France; ^3^UFR Sciences et Techniques, 6 avenue Victor Le Gorgeu, 29238 Brest Cedex 3, France; ^4^Institut Universitaire de Cardiologie et de Pneumologie de Québec, Université de Laval, 2725 chemin Ste-Foy, Québec, QC, Canada G1V 4G5; ^5^Département de Cardiologie, Centre Hospitalo-Universitaire de Brest, boulevard Tanguy Prigent, 29200 Brest, France

## Abstract

Increased sugar consumption, especially fructose, is strongly related to the development of type 2 diabetes (T2D) and metabolic syndrome. The aim of this study was to evaluate long term effects of fructose supplementation on Wistar rats. Three-week-old male rats were randomly divided into 2 groups: control (C; *n* = 14) and fructose fed (FF; *n* = 18), with a fructose enriched drink (20–25% w/v fructose in water) for 21 weeks. Systolic blood pressure, fasting glycemia, and bodyweight were regularly measured. Glucose tolerance was evaluated three times using an oral glucose tolerance test. Insulin levels were measured concomitantly and insulin resistance markers were evaluated (HOMA 2-IR, Insulin Sensitivity Index for glycemia (ISI-gly)). Lipids profile was evaluated on plasma. This fructose supplementation resulted in the early induction of hypertension without renal failure (stable theoretical creatinine clearance) and in the progressive development of fasting hyperglycemia and insulin resistance (higher HOMA 2-IR, lower ISI-gly) without modification of glucose tolerance. FF rats presented dyslipidemia (higher plasma triglycerides) and early sign of liver malfunction (higher liver weight). Although abdominal fat weight was increased in FF rats, no significant overweight was found. In Wistar rats, 21 weeks of fructose supplementation induced a metabolic syndrome (hypertension, insulin resistance, and dyslipidemia) but not T2D.

## 1. Introduction

Type 2 diabetes (T2D) is a metabolic disease defined by a fasting hyperglycemia frequently related to the progressive development of a resistance towards insulin as well as a reduced glucose tolerance. According to the World Health Organization (WHO), the prevalence of this disease is rapidly expanding [1]; diabetes is therefore considered epidemic [2]. Another metabolic disease strongly correlated with T2D is the metabolic syndrome. Indeed, according to the WHO, metabolic syndrome is defined as insulin resistance and/or T2D associated with two or more of the following symptoms: hypertension, elevated plasma triglycerides, low HDL cholesterol, obesity, and higher urinary albumin excretion rate [3]. The rapid increase in diabetes prevalence is strongly related to changes in our lifestyle including reduced physical activity and changes in our diet [1]. Increased sugar consumption, and more specifically fructose consumption (a common sweetener used in soft drink), is strongly related to the development of T2D as well as metabolic syndrome parameters (hypertension, dyslipidemia, and obesity) [4, 5]. Modification of oxidative status is also known to be part of T2D consequence, as 12 weeks of a human-like fructose enriched diet progressively induced antioxidant enzyme activity levels [6]. However, such variation mechanisms still remain to be investigated.

Tappy and Le (2010) [5] highlighted the lack of studies close to the actual human consumption of fructose: around 50 g/day in Europe for a man (up to around 70 g/day in the United States of America), mainly consumed in sweet beverage starting as early as 6 years or even 4 years in the United Kingdom [4, 5]. Most animal studies on fructose enriched diet use Sprague-Dawley rats [7–12]. However, Sprague-Dawley rats are known to be more sensitive towards the development of T2D and are therefore less representative of human population [7, 13]. The majority of studies on fructose effects were performed over a period between 8 and 12 weeks long [6, 7, 14–17], using mainly high quantity of fructose (60–66% of fructose in the chow) [7, 8, 17]. Recently, some authors had begun to define more realistic diets using Sprague-Dawley rats [18] as well as Wistar rats [19]. However, these studies only adapted the quantity of fructose used and the method of ingestion to be more representative of human consumption, but the age at the beginning of the diet was not. Our study proposes addressing this issue using a reality-like fructose enriched diet starting shortly after weaning. 12-week human-like fructose enriched diet is already known to induce the early signs of T2D in Wistar rats [4]. Longer term effects of such a diet are still left to investigate in Wistar rats.

The aim of this study was thus to evaluate the long term impacts of a reality-like fructose enriched diet on various parameters such as bodyweight, fasting glycemia, insulin resistance, and plasma lipids levels. Novel approaches were used including regular monitoring of the systolic blood pressure, as well as mRNA studies on antioxidant enzymes.

## 2. Methods

### 2.1. Animals

All experiments were approved by the French Ethical Committee for Animal Care and Use number 74 and were authorized on August 11, 2015, by the French “Ministère de l'Éducation Nationale, de l'Enseignement Supérieur et de la Recherche” under the number APAFIS 773.

32 male Wistar rats (Janvier Labs), 3 weeks old (weight under 49 g, Janvier Labs, Le Genest Saint Isle, France), were received on the same day and were housed individually in a light (12 h:12 h light/dark cycle) and temperature (20°C ± 1°C) controlled animal facility. Rats were all fed with a standard chow (Kliba Nafag, M/R Maintenance, metabolizable energy: 3.15 kcal/g). Rats were randomly assigned to one of the two following groups: FF (*n* = 18) and C (*n* = 14). FF had a fructose enriched drink (20% w/v between the ages of 3 weeks and 9 weeks and 25% w/v between the ages of 10 weeks and 24 weeks), while the other group C had tap water. Fructose enriched drinks were changed every couple of days; water bottles were sterilized every week. Bodyweight (bw) was measured weekly. To ensure a constant supply of fructose (quantity of fructose ingested/bodyweight), fructose percentage in the drinking was increased after the fast growing period, that is to say, starting at the age of 10 weeks. Fructose (Vivis, Saint Genis Laval, France) had an energy value of 4 kcal/g.

### 2.2. Sampling

Rats were anesthetized with Ketamine (Ketamine 100, Virbac, 80 mg·kg^−1^)/Xylazine (Rompun 2%, Bayer, 12 mg·kg^−1^) injected intramuscularly (into the left back leg). Blood was collected intraventricularly in 2 mL sampling tubes (precoated with EDTA). Plasma was obtained after a 15 min centrifugation at 1000*g*. Heart, liver, and soleus muscle were collected and dried from any residual blood and then weighed. Abdominal fat (retroperitoneal and epididymal excluding subcutaneous fat) was also collected and weighed. Plasma and organs were immediately frozen in liquid nitrogen and then stored at −80°C for further analysis.

Heart weight (% bodyweight) was calculated as follows:

(1) $$\begin{array}{c} \text{heart weight}\left(\;\%\text{ bw}\;\right)=\frac{\text{heart weight}\left(g\right)}{\text{body weight}\left(g\right)}\times 100. \end{array}$$

Soleus weight and liver weight as well as abdominal fat weight (% bodyweight) were calculated using the same formula.

### 2.3. Metabolic Measurements

After a 15 h fast, fasting glucose was measured in blood collected by a single prick onto the mandibular veins (allowing only one drop to come off) using a glucometer (Accu-Chek Performa, Roche, Meylan, France) [20].

Oral glucose tolerance was performed three times (at the ages of 9, 15, and 23 weeks) as described in Dupas et al. [6]. Area under curve was then calculated to estimate glucose tolerance.

During OGTT, blood samples were collected in 300 *μ*L Microvette tubes precoated with He-Li (Sarstedt, Nümbrecht, Germany). Following the manufacturer recommendation, plasma was obtained after 5 min at 2000*g* centrifuge. Plasma from OGTT blood sample was obtained after a 5 min centrifugation at 2000*g* and then frozen and stored at −80°C before further analysis. Insulin concentration was evaluated on those plasma samples using ELISA methods (Rat Insulin ELISA, ALPCO, Eurobio, Courtaboeuf, France).

From OGTT and insulin concentration data, two insulin resistance and sensitivity indicators were determined: the Homeostatic Model Assessment for Insulin Resistance (HOMA 2-IR) and then the Insulin Sensitivity Index for glycemia (ISI-gly). HOMA-IR was calculated using a HOMA 2-IR calculator software [21]. The software is available at https://www.dtu.ox.ac.uk/homacalculator, Oxford University, and uses OGTT data; that is, fasting insulinemia and fasting glycemia were taken at *t* = 0 min (before the ingestion of a high dose of glucose (1 g/kg bw)). ISI-gly was calculated as follows [22], with AUC for area under curve:

(2) $$\begin{array}{c} \text{ISI-gly}=\frac{2}{\left[\left(\text{AUC glycemia}\times \text{AUC insulin}\right)+1\right]}. \end{array}$$

### 2.4. Systolic Blood Pressure

Systolic blood pressure was measured regularly (every 2-3 weeks), using a tail cuff blood pressure system (Model 29 pulse amplifier with tail cuff sensor and adapted rodent restrainer, iitc incorporated). To avoid any stress, rats were acclimated to the blood pressure system for 4 days before measurement.

### 2.5. Blood Biochemistry

Blood chemistry measures were performed on a Konelab 20 (Thermo Scientific) using adapted kit for, aspartate aminotransferase activity (ASAT) (Biomérieux), alanine aminotransferase activity (ALAT) (Biomérieux), creatinine (Jaffé method, Fisher Brahms), albumin (bromocresol green method, Biomérieux), nonesterified fatty acid (NEFA) (Wako), triglycerides (PAP methods, Biomérieux), total cholesterol (Cholesterol RTU, Biomérieux), and urea (Thermo Fisher Scientific). The ASAT/ALAT ratio was then calculated.

Different molar ratios were calculated as a part of the lipids levels evaluation: NEFA to cholesterol ratio [23], NEFA to albumin ratio [24], and cholesterol to triglycerides ratio [25].

Theoretical creatinine clearance was calculated using the Cockcroft and Gault formula [26] that has been already used in rat model [27].

(3) Theoretical creatinine clearanceml/min=140−ageyears∗bodyweightkgcreatininemg/dL∗72.

### 2.6. Hepatic Histology

Small portion of the liver was sampled and immediately put in a fixative solution (Bouin's solution) for at least 48 h. Samples were then embedded in paraffin and transverse sections of 5 *μ*m were then cut. The severity of hepatic complications was assessed by eosin/hematoxylin coloration.

### 2.7. Antioxidant Enzyme Activities

300 mg of either left gastrocnemius or left ventricle was homogenized in a 4°C Tris-EDTA buffer (75 mM/5 mM) with an ultrathurax. The homogenate was centrifuged 10 min at 100*g*, 4°C; after that the supernatant was centrifuged for 10 min at 12000*g*, 4°C. The supernatant was kept at −80°C until analysis. Proteins levels were measured using the BCA method (Interchim Uptima Protein Quantification kit). Automated plate reader was used for the analysis (SAFAS, Monaco).

Superoxide dismutase (SOD), catalase (CAT), and glutathione peroxidase (GPx) activities level were measured as previously described in Dupas et al. [6].

### 2.8. RNA Isolation for RT-PCR

Total RNA was isolated from the left ventricle of control and fructose fed rats (*n* = 6) using the Nucleospin RNA Mini kit (Macherey-Nagel, France) according to a manufacturer's protocol adapted for fibrous tissue. Briefly, up to 30 mg of tissue previously ground in liquid nitrogen was homogenized 2 × 15 s with an ultrathurax (VWR, France) in 350 *μ*L of RA1 buffer containing 3.5 *μ*L of *β*-mercaptoethanol and Triton X-100 (SIGMA Aldrich, France). An enzymatic lysis was then performed with Proteinase K at room temperature for 10 min followed by 10 min at 55°C. After centrifugation at 8,000*g* for 1 min, the supernatants were mixed to absolute ethanol and transferred into Nucleospin columns. The following RNA extraction steps, which included a DNAse treatment, were performed according to the procedure described in the kit handbook. RNA was eluted with 40 *μ*L of DNAse/RNAse-free water and stored at −80°C. RNA concentrations were measured with a NanoDrop ND 1000 (Thermo Scientific, France) and their purity was assessed using OD260/OD280 ratios. Their integrity was also checked by an electrophoresis on 1.5% agarose gel.

### 2.9. Quantification of Gene Expression by Real-Time Reverse Transcriptase-PCR (RT-PCR)

Real-Time RT-PCR was used to quantify and compare the mRNA levels of different genes in the heart of standard and FF rats. Total RNA of each sample (1000 ng) was reverse transcribed with the qScript™ cDNA synthesis kit (QUANTA BioSciences, VWR, France) containing a mix of oligo(dT) and random hexamers. All cDNA were then diluted 10-fold for PCR experiments, which was realized with a 7500 Fast Real-Time PCR apparatus (Applied Biosystems, Thermo Fisher Scientific, France). Target genes were amplified and quantified by SYBR® green incorporation (EurobioGreen® Mix qPCR 2x Lo-Rox) with the following primers: 18S rRNA, forward: 5′ AGA AAC GGC TAC CAC ATC CAA 3′, reverse: 5′ CAA TTA CAG GGC CTC GAA AGA 3′;* sod1*, forward: 5′ATT AAC TGA AGG CGA GCA TGG 3′, reverse: 5′ TCC AAC ATG CCT CTC TTC ATC 3′;* sod2*, forward: 5′ TGG CTT GGC TTC AAT AAG GAG 3′, reverse: 5′ AAG ATA GTA AGC GTG CTC CCA 3′;* cat*, forward: 5′ CAT GAA TGG CTA TGG CTC ACA 3′, reverse: 5′ AAG TCT TCC TGC CTC TTC AAC 3′;* gpx1*, forward: 5′ TGC AAT CAG TTC GGA CAT CAG 3′, reverse: 5′ TTC ACC TCG CAC TTC TCA AAC 3′. The cycling conditions consisted of a denaturing step at 95°C for 2 min, followed by 40–45 cycles of amplification (denaturation: 95°C for 5 s; annealing/extension step: 60°C for 30 s). Finally, a melting curve program was carried out from 60°C to 95°C with a heating rate of 0.1°C per s, showing a single product with a specific melting temperature for each gene and sample evaluated.

Standard curves were established to determine and compare the transcription level of the different target genes in the two experimental groups. To obtain these curves, all target genes were first amplified from a pool of RT products prepared with both standard and FF samples. PCR products were purified after electrophoretic separation on a 1.5% agarose gel using the Nucleospin gel and PCR Clean-Up® kit (Macherey-Nagel). PCR products were then quantified using a Nanodrop spectrophotometer before proceeding to serial dilution from 10 pg/*μ*L to 0.001 fg/*μ*L. A seven-point standard curve was used to determine the PCR efficiency of each primer pair (between 80 and 100%) and to determine the transcription level of the different genes in all samples. Each gene was amplified in a single run from triplicates of standard points and samples. Quantification was normalized using 18S rRNA as housekeeping gene and all mRNA levels were expressed as a ratio = target gene/18S rRNA.

### 2.10. Statistics

All results are expressed in mean ± standard error of mean (SEM). All statistics were performed using Statistica v. 12 software (StatSoft, France). Normality of study population was tested using the Shapiro-Wilk test. Adapted tests were then performed (Student's *t*-test, Mann–Whitney *U* test, and ANOVA for repeated measures). ANOVA were followed by a post hoc test (HSD).

## 3. Results

### 3.1. Systolic Blood Pressure and Heart Volume

**Figure 1:**
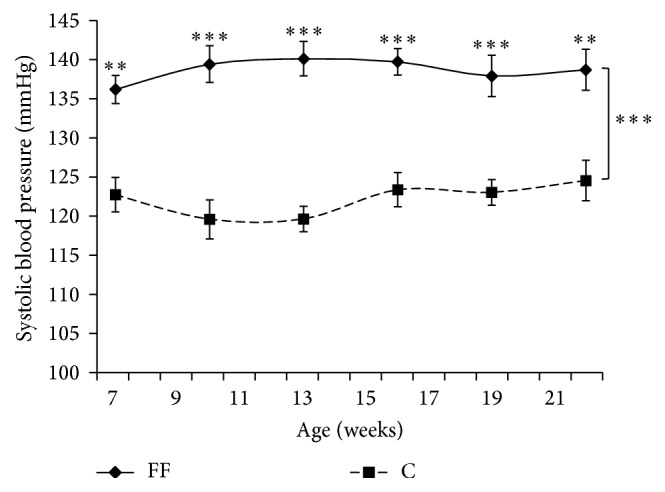
Effects of fructose supplementation on systolic blood pressure. Systolic blood (mmHg) is represented as a function of age (weeks). Groups: FF: fructose fed; C: control. Statistical values: ^*∗∗*^*p* < 0.01;  ^*∗∗∗*^*p* < 0.001.

Systolic blood pressure was measured regularly (Figure 1). FF had a higher systolic blood pressure than C (*p* < 0.001). The 4 weeks of fructose enriched diet (i.e., rats were 7 weeks old) increased systolic blood pressure in FF (FF: 136.3 ± 1.8 versus C: 122.7 ± 2.2 mmHg). Systolic blood pressure was statistically higher in FF during the whole study.

### 3.2. Bodyweight and Organs Weight

**Figure 2:**
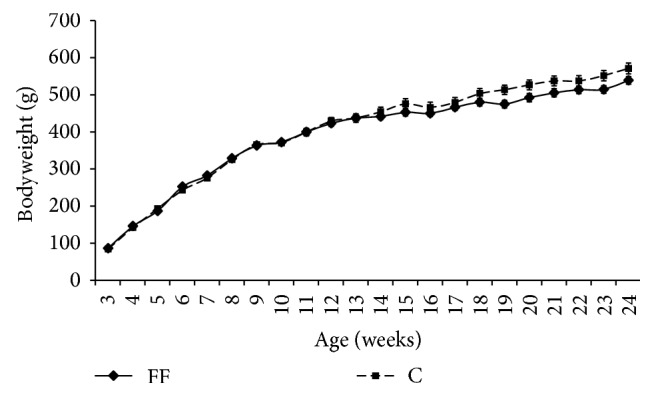
Effects of fructose supplementation on bodyweight. Bodyweight (g) is represented as a function of age (weeks). Groups: FF: fructose fed; C: control.

Rats bodyweight (bw) was evaluated weekly (Figure 2). Throughout the whole duration of the study, no statistical difference has been found between FF and C bodyweight.

Heart, abdominal fat, and liver weight are summarized in Table 1. No statistical difference was found between FF and C heart weight. However FF rats had a higher abdominal fat weight than C rats (FF: 5.04 ± 0.24 versus C: 3.86 ± 0.27  (% bw),  *p* < 0.01) and FF presented a heavier liver than C (FF: 2.68 ± 0.061 versus C: 2.39 ± 0.056 (% bw), *p* < 0.01).

### 3.3. Fasting Glycemia, Glucose Tolerance, and Insulin Resistance

**Figure 3:**
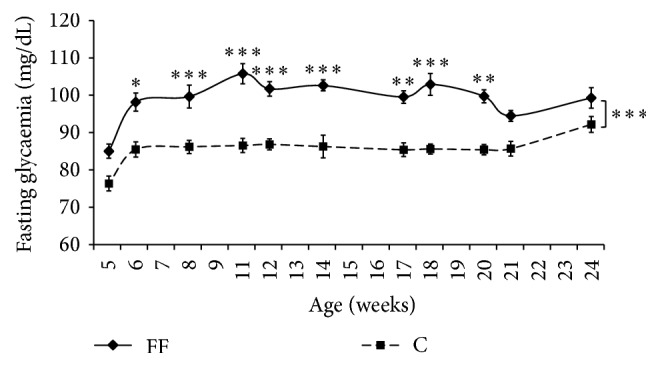
Effects of fructose supplementation on fasting glycemia. Fasting glycemia (mg/dL) is represented as a function of age (weeks). Groups: FF: fructose fed; C: control. Statistical values: ^*∗*^*p* < 0.05,  ^*∗∗*^*p* < 0.01, and  ^*∗∗∗*^*p* < 0.001.

Fasting glycemia was measured regularly; FF had a higher fasting glycemia than C for the entire duration of the study (*p* < 0.001) (Figure 3). More precisely, at the age of 5 weeks (i.e., after 2 weeks of fructose enriched diet) fasting glycemia was not different between FF and C rats (FF: 85.4 ± 1.9 versus C: 76.7 ± 2.0 mg/dL). However 3 weeks of fructose enriched diet (age of 6 weeks) was sufficient to induce a significant increase (FF: 98.6 ± 2.4 versus C: 85.8 ± 2.0 mg/dL, *p* < 0.05).

Oral glucose tolerance test was performed three times: at the ages of 9, 15, and 23 weeks, from these measurements, area under curve (AUC) was calculated (Table 2). No statistical difference was found between FF and C AUC, although a trend for FF to have higher AUC than C was found at the ages of 9 weeks (*p* = 0.077) and 23 weeks (*p* = 0.082).

Concomitantly to OGTT measurements, insulin levels were studied (Table 2) and enabled the calculation of HOMA 2-IR and ISI-gly. HOMA 2-IR is higher in FF rats than in C rats at the ages of 9, 15, and 23 weeks (9 weeks: FF 2.83 ± 0.26 versus C 1.52 ± 0.28, *p* < 0.01). ISI-gly is lower in FF than C rats at the ages of 9 weeks (FF: 0.117 ± 0.009 versus C: 0.175 ± 0.011, *p* < 0.001) and 23 weeks (FF: 0.079 ± 0.013 versus C: 0.109 ± 0.01, *p* < 0.01). However no statistical difference was found at the age of 15 weeks. Matsuda index could not be used because some insulin values at the beginning of the OGTT were too low to enable its calculation.

### 3.4. Theoretical Creatinine Clearance, Plasma ASAT/ALAT Ratio, Albumin, Urea, and Lipids Levels

Table 1 summarizes the results at the age of 23 weeks. Moreover, it can be noticed that FF had higher triglycerides levels than C (1393.9 ± 113.7 versus 836.0 ± 67.0 mg/L, *p* < 0.001); FF also had a lower total cholesterol/triglycerides ratio (1.42 ± 0.09 versus 2.33 ± 0.23, *p* < 0.001). In addition FF had a lower plasma urea than C (300.2 ± 13.3 versus 409.9 ± 18.6 mg/L, *p* < 0.001). No other statistical difference was found upon the other measured parameters (ASAT/ALAT, theoretical creatinine clearance, NEFA, total cholesterol, and NEFA/albumin).

### 3.5. Hepatic Histology

**Figure 4:**
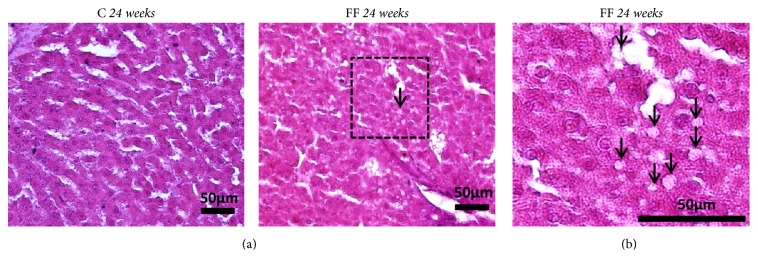
Hepatic histology was assessed using eosin/hematoxylin coloration. Small lipids droplet can be found at the age of 24 weeks (arrow) (a). Enlargement of the frame can be found (b).

Despite the presence of few microvesicular lipid droplets in the liver of FF rats at the age of 24 weeks, the presence of nonalcoholic fatty liver disease (NAFLD) is not confirmed (Figure 4). Only 4 out of 17 FF rats presented between 10 and 20 microvesicular lipid droplets on the whole observed section.

### 3.6. Antioxidant Enzymes: Activities and mRNA Levels

**Figure 5:**
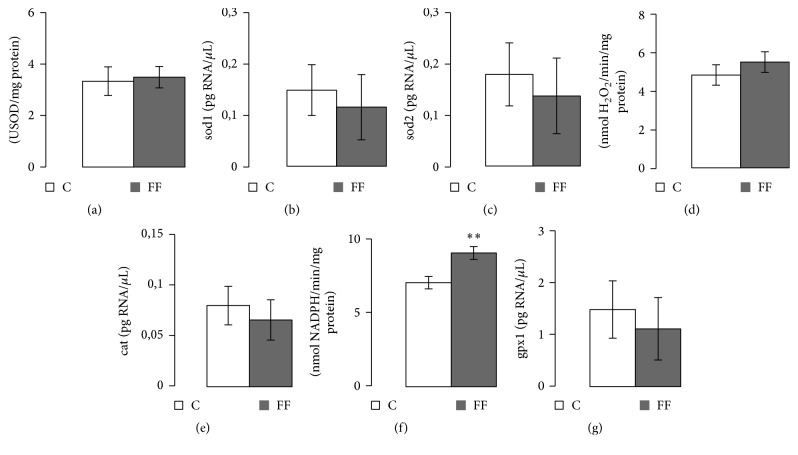
Effects of fructose supplementation on antioxidant enzymes activities and their corresponding mRNA levels in left ventricle. (a) SOD activity (USOD/mg protein), (b)* sod1* mRNA levels (pg/*μ*L), (c)* sod2* mRNA levels (pg/*μ*L), (d) CAT activity (nmol of H2O2/min/mg protein), (e)* cat* mRNA levels (pg/*μ*L), (f) GPx activity (nmol NADPH/min/mg protein), and (g)* gpx1* mRNA level (pg/*μ*L). Groups: FF: fructose fed; C: control. Statistical values: ^*∗∗*^*p* < 0.01.

**Figure 6:**
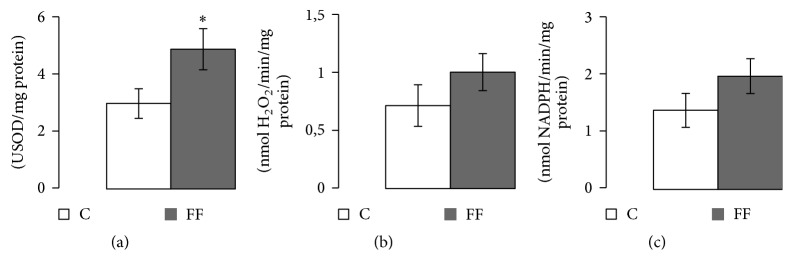
Effects of fructose supplementation on antioxidant enzymes activities in left gastrocnemius. (a) SOD activity (USOD/mg protein), (b) CAT activity (nmol of H2O2/min/mg protein), and (c) GPx activity (nmol NADPH/min/mg protein). Groups: FF: fructose fed; C: control. Statistical values: ^*∗*^*p* < 0.05.

Three antioxidant enzyme activities were measured (SOD, CAT, and GPx) in two muscles: the left ventricle and the gastrocnemius. In addition, in the left ventricle* sod1*,* sod2*,* cat*, and* gpx1* mRNA quantities were measured. In the left ventricle (Figure 5), while SOD and CAT activity levels, as well as their corresponding mRNA levels, remained unchanged, GPx activity was increased in FF rats (9.06 ± 0.43 versus 7.05 ± 0.42 nmol NADPH/min/mg protein, *p* < 0.01). However* gpx1* mRNA levels remained similar in both groups. In the gastrocnemius (Figure 6), only SOD activity was increased in FF rats (4.92 ± 0.72 versus 3.01 ± 0.52 USOD/mg protein, *p* < 0.05). The other two studied enzymes showed no significant variation.

## 4. Discussion

While being well studied in Sprague-Dawley rats [7–12], long term effects of fructose enriched diet are not in Wistar rats. With their more active behaviour and higher metabolic rate [7, 15], Wistar rats are less sensitive towards T2D than Sprague-Dawley rats. Wistar rats are thus more representative of human population [7, 13]. The originality of the diet we used was the age at which it started. Our fructose enriched diet was introduced in drinking water just after weaning, to better represent human consumption (50 g/day, mostly in sweet drink, starting in early childhood) [5].

This study showed that a fructose enriched diet induces progressively a higher fasting glycemia after 3 weeks (Figure 3). This hyperglycemia was not associated with a reduced glucose tolerance even after 6, 12, or 20 weeks of fructose enriched diet. Indeed, AUC calculated from OGTT are similar with or without the fructose enriched diet (Table 2). Surprisingly, fructose rapidly induced an insulin resistance as well as a reduced insulin sensitivity, respectively, shown with variations of HOMA 2-IR [21] and ISI-gly [22]. Hepatic insulin sensitivity is provided by HOMA 2-IR [28] as this marker indicates insulin resistance for a value higher than 1.85 [29]. Six weeks of fructose supplementation was sufficient to induce insulin resistance in FF rats with HOMA 2-IR 1.5-fold the cut-off value. Furthermore, after 12 and 20 weeks of fructose enriched diet HOMA 2-IR values were, respectively, 2.13- and 2.4-fold the cut-off value. However C rats also developed an insulin resistance at age 23 weeks as their HOMA 2-IR value reached 2.44 ± 0.46, thus adding sedentarity to fructose enriched diet and age as factors that led to insulin resistance in Wistar rats. On the other hand ISI-gly is considered to be a whole body/peripheral Insulin Sensitivity Index [30], lower ISI-gly indicating reduced insulin sensitivity. In our study 6 weeks of fructose supplementation was sufficient to significantly reduce ISI-gly (FF: 0.117 ± 0.009 versus C: 0.175 ± 0.011, *p* < 0.01). Surprisingly, at the age of 15 weeks (i.e., after 12 weeks of fructose supplementation) ISI-gly was similar in both groups. Nonetheless after 20 weeks of fructose enriched diet, FF rats had a lower ISI-gly than the control rats. Combined HOMA 2-IR and ISI-gly indicate that after 6 weeks of fructose supplementation rats became insulin resistant, which is consistent with a previous finding [6]. They remained insulin resistant after 12 weeks but in a weaker manner as their insulin sensitivity was not additionally decreased. At the age of 23 weeks, all rats were insulin resistant. However C rats had a lower HOMA 2-IR and were more sensitive to insulin than FF. In summary, fructose enriched diet rapidly induced insulin resistance in Wistar rats; meanwhile age and sedentarity resulted in insulin resistance in a slower manner. Lozano et al. (2016) studies, using a similar diet, are in accordance with ours. They suggest that fructose enriched diet only speeds up the natural development of insulin resistance in Wistar rats [19].

Another symptom was observed in our study, a high blood pressure in FF rat. Systolic blood pressure was evaluated throughout the study (Figure 1). Fructose enriched diet rapidly induced a persistent hypertension for the rest of the protocol. The increase in blood pressure might have been progressive; however for technical reasons we were unable to evaluate systolic blood pressure in rats younger than 7 weeks due to their small size. Fructose-induced hypertension is already a well-established fact. However its mechanism remains unknown [19, 20]. A minimal consumption of fructose is needed, as previous study showed that 6 weeks of 10% of fructose in drinking water is not sufficient to induce hypertension [16]. In our study, fructose supplementation was not associated with a cardiac hypertrophy as shown by the similar heart weight in FF and C (Table 1). Renal function was also studied as a possible source of hypertension. For this purpose theoretical creatinine clearance was calculated using the Cockcroft and Gault formula, which was previously validated in rats [4, 11]. Theoretical creatinine clearance was not modified by fructose supplementation precluding the hypothesis of renal failure as a cause of hypertension in our FF rats.

On the contrary of what was previously described [6], in this study fructose supplementation did not generate weight gain (Figure 2) but still induced a change in body composition enhancing abdominal fat weight (Table 1). Furthermore, fructose supplementation increases energy intake (S1 Fig in Supplementary Material available online at https://doi.org/10.1155/2017/2494067)  (calculated from food and drink intake (S1 and S2 Table)). It can also been noticed that fructose supplementation reduced food intake which is consistent with previous studies on fructose supplementation [31].

Another morphological observation was the increase in liver weight during fructose supplementation, a possible first sign of fatty liver [32], although the liver function was not modified (ASAT/ALAT remained unchanged). The presence of NAFLD was not confirmed. Previous studies showed that high fructose diet can lead to liver dysfunction as well as NAFLD, even though it is not a constant fact [5, 19, 33]. The dose of fructose seems to be an important factor in the development of NAFLD; higher dose up to 60% of fructose in the chow seems to lead to its development [5, 33], while reality-like dose of fructose does not (Figure 4) [19]. Using our reality-like fructose enriched diet, only a few rats developed small lipids droplets (10 to 20 lipids droplets per section) in the liver after 21 weeks (shortly over 5 months) of fructose enriched diet. This is in accordance with a recent study using a similar fructose enriched diet, although started at an older age than in our experiment [19]. These authors showed that even after 8 months of fructose enriched diet, Wistar rats did not develop NAFLD. In addition, FF rats presented a higher plasma urea than C rats (Table 1). Plasma urea is derived from plasma uric acid. In the liver, fructose is phosphorylated is fructose-1-phosphate leading to ATP hydrolysis to AMP and then to the formation of uric acid. Thus higher fructose levels lead to higher uric acid levels [34], another well-established risk factor for the development of T2D [35].

Metabolic syndrome signs include insulin resistance, hyperglycemia, and dyslipidemia. Previous studies with shorter duration of fructose enriched diet (between 3 and 12 weeks) were inconsistent about lipid levels variations [6, 7, 14, 36]. In our studies, 21 weeks of fructose supplementation induced higher triglycerides levels and a lower total cholesterol/triglycerides ratio (Table 1) which may predict a higher presence of small dense LDL [25] and a higher cardiovascular disease risk [37].

Regarding antioxidant enzyme activities, various studies on T2D and hyperglycemia showed contrasting results [38–41]. In our study, different patterns were found in the 2 studied tissues: heart and gastrocnemius. In the gastrocnemius, SOD activity levels were increased, while the other antioxidant enzymes levels (CAT and GPx) remained unchanged (Figure 6). However, in the left ventricle, GPx levels were enhanced with fructose supplementation (Figure 5). Previous use of the same fructose enriched diet but only for 12 weeks had already shown a progressive increase in SOD and CAT activities levels without change in GPx activity in rat left ventricle. This difference may be age-related leading to a different physiological adaptation to a longer diet (21 weeks). It has already been shown that T2D as well as metabolic syndrome increased the levels of reactive oxygen species (ROS), like superoxide (O_2_^−∙^) [42, 43]. If not detoxified by antioxidant enzymes, ROS can induce dangerous consequences on the metabolism such as lipid peroxidation or DNA mutation [42, 43]. O_2_^−∙^ is detoxified in H_2_O_2_ by the SOD, while H_2_O_2_ is detoxified by either the CAT or the GPx [44]. Furthermore, a higher level of O_2_^−∙^ can rapidly bound to nitric oxide (NO) to produce nitric peroxide (ONOO−), another oxidant molecule harmful to vascular function [45]. Nitric oxide (NO) is produced mainly by the endothelial nitric oxide synthase (eNOS), also present in the heart [46]. One of the ONOO− harmful consequences is the inhibition of various antioxidant enzymes including SOD and CAT [47], an effect that may explain the fact that SOD increased activity levels were only found using shorter diet duration. Another potential consequence of increased ONOO− production is a vascular dysfunction [45]. Further studies on vascular function are needed to confirm or infirm our hypothesis.

In order to investigate the origin of antioxidant enzyme levels modifications, corresponding mRNA levels were studied in the left ventricle. No variation was observed, which may indicate that antioxidant enzyme activities are regulated only at a posttranscriptional level. Our results are in contradiction with the few existing studies on antioxidant enzymes mRNA levels, which showed decreased mRNA levels for both SOD and CAT and GPx after 3 weeks of fructose enriched diet in Wistar rats [48]. The longer duration of the study may be an explanation, as the cellular metabolism needs a longer adaptation period.

Although this study clearly shows the development of metabolic disorders following fructose consumption in rats, some limitations can be cited. Indeed, in addition to indirect measurements of LDL, direct measurements of both HDL and LDL cholesterol would have helped to clearly define the dyslipidemia [37]. To the same extent, a direct measurement of uric acid associated with the plasma urea measurements that have been already realized may have added stronger evidence towards the development of T2D [34, 35]. Measurements of inflammation markers such as TNF-*α* and O_2_^−∙^ would have helped to complete the inflammation status and might have helped to better interpret the antioxidant enzymes levels [42, 43]. Unfortunately blood samples were not sufficient to determine all of the above.

## 5. Conclusion

In Wistar rats, a 21-week-long fructose supplementation (20–25% w/v) started right after weaning rapidly induced a chronic hypertension, dyslipidemia, and fasting hyperglycemia associated with insulin resistance. Overweight as well as NAFLD was not associated with these findings. According to the WHO criteria, our 21-week fructose enriched diet induced a metabolic syndrome and surprisingly did not result yet in T2D. Further studies may be needed to elucidate the mechanisms by which fructose supplementation promotes these changes.

## Supplementary Material

S1 Fig. Effects of fructose consumption on food (A), drink (B) and energy intake (C). Energy intake (kcal/day/kg of bodyweight) was calculated from the food (kcal/day) and drink (mL/day) intake.
S1 Table. Raw data for drink intake (mL/day).
S2 Table Raw data for food intake (g/day).

## Figures and Tables

**Table 1:** Effects of fructose supplementation on abdominal fat weight, liver weight, heart weight, theoretical creatinine clearance, and plasma urea, ASAT, ALAT, albumin, and lipid levels. FF: fructose fed; C: control.

	C *n* = 14	FF *n* = 17	*p*
Heart weight (% bw)	0.274 ± 0.005	0.281 ± 0.006	0.37
Abdominal fat weight (% bw)	3.86 ± 0.27	5.04 ± 0.24	0.0029
Liver weight (% bw)	2.39 ± 0.06	2.68 ± 0.06	0.0016
Plasma urea (mg/L)	409.9 ± 18.6	300.2 ± 13.3	0.0001
ASAT/ALAT	2.01 ± 0.12	2.42 ± 0.18	0.36
Theoretical creatinine clearance (mL/min)	2.13 ± 0.07	2.08 ± 0.05	0.60
NEFA (*µ*mol/L)	532.6 ± 15.7	627.5 ± 50.1	0.34
Triglycerides (mg/L)	836.0 ± 67.0	1393.9 ± 113.7	0.0004
Total cholesterol (mg/L)	779.6 ± 37.2	795.8 ± 21.4	0.69
NEFA/total cholesterol (molar ratio)	0.274 ± 0.014	0.307 ± 0.024	0.27
NEFA/albumin (molar ratio)	1.071 ± 0.033	1.208 ± 0.093 *n* = 16	0.61
Total cholesterol/triglycerides (molar ratio)	2.33 ± 0.23	1.42 ± 0.09	0.0005

**Table 2:** Effects of fructose supplementation on oral glucose tolerance test (shown using the area under curve), HOMA 2-IR, and ISI-gly. Each parameter was measured at the ages of 9, 15, and 23 weeks (*n* = 14 for FF and *n* = 12 for C at all ages). FF: fructose fed; C: control.

	9 weeks			15 weeks			23 weeks		
	C *n* = 12	FF *n* = 14	*p*	C *n* = 12	FF *n* = 14	*p*	C *n* = 12	FF *n* = 14	*p*
OGTT (area under curve)	7.18 ± 0.12	7.49 ± 0.12	0.078	6.98 ± 0.12 *n* = 12	7.02 ± 0.10	0.83	6.80 ± 0.18	7.20 ± 0.13	0.082
HOMA 2-IR	1.52 ± 0.28	2.83 ± 0.26	0.0022	1.79 ± 0.29 *n* = 12	3.94 ± 0.59	0.0048	2.44 ± 0.46	4.43 ± 0.32	0.0014
ISI-gly	0.175 ± 0.011	0.117 ± 0.009	0.0006	0.118 ± 0.013	0.096 ± 0.013	0.36	0.109 ± 0.010	0.079 ± 0.008	0.0016
