# Supplementary material for: Metabolic Syndrome and Hypertension Resulting from Fructose Enriched Diet in Wistar Rats
Source: Biomed Res Int. 2017 Apr 11;2017:2494067. doi: 10.1155/2017/2494067 (PMC5405603; doi:10.1155/2017/2494067)
Supplement: Supplementary file 1 — S1 Fig. Effects of fructose consumption on food (A), drink (B) and energy intake (C). Energy intake (kcal/day/kg of bodyweight) was calculated from the food (kcal/day) and drink (mL/day) intake. S1 Table. Raw data for drink intake (mL/day). S2 Table Raw data for food intake (g/day). [file 2494067.f1.zip › Supplementary files pdf/Table S2.pdf]

		Drink intake (mL/day)																			
	Age (weeks)	4	5	6	7	8	9	10	11	12	13	14	15	16	17	18	19	20	21	22	23
up	Rat																				
, 24weeks		*n=14*	*n=14*	*n=14*	*n=14*	*n=14*	*n=14*	*n=14*	*n=14*	*n=14*	*n=14*	*n=14*	*n=14*	*n=14*	*n=14*	*n=14*	*n=14*	*n=14*	*n=14*	*n=14*	*n=14*
	C1	33.7	45.3	51.1	52.0	47.4	50.5	49.0	42.7	47.7	44.7	43.9	52.5	40.5	45.2	48.8	51.8	50.0	51.2	44.9	48.0
	C2	31.6	42.7	49.1	46.7	42.7	46.8	38.7	41.4	40.1	44.8	41.9	30.6	32.9	33.4	44.0	41.2	38.1	43.6	39.2	41.4
	C3	33.6	34.1	47.7	43.6	38.0	41.6	36.1	35.5	38.4	41.6	41.1	35.7	31.8	26.6	32.0	30.9	33.1	33.6	33.3	33.4
	C4	34.1	47.1	61.1	46.4	47.4	51.3	42.8	44.5	46.9	47.6	49.1	49.2	35.0	29.2	41.1	42.6	39.8	45.1	38.5	41.8
	C5	29.7	40.5	50.4	47.6	44.0	53.1	47.3	45.0	48.2	51.9	39.7	48.4	41.7	40.2	39.1	35.7	40.5	45.1	45.4	45.2
	C6	27.3	36.4	35.0	32.8	29.4	36.2	30.4	30.6	31.3	35.2	35.7	28.8	17.2	24.7	29.1	33.8	34.9	30.8	28.4	29.6
	C7	31.0	44.9	48.6	42.4	41.5	46.9	39.9	45.3	44.8	46.9	40.8	45.3	30.7	31.1	32.1	32.3	35.7	40.1	36.7	38.4
	C8	32.1	39.9	46.9	42.9	38.8	47.8	41.8	42.5	40.4	45.4	37.4	48.3	37.6	32.8	39.3	40.4	43.5	43.4	38.0	40.7
	C9	24.7	39.3	43.2	40.1	34.2	44.1	37.5	35.2	41.0	42.3	43.2	46.0	37.2	34.5	37.4	36.4	41.3	37.3	23.8	30.5
	C10	29.5	41.3	50.9	46.4	41.2	47.4	41.7	40.2	41.9	47.6	37.2	48.0	44.3	38.3	40.3	42.1	47.3	39.0	40.9	40.0
	C11	28.3	41.5	42.4	42.5	39.5	47.8	45.3	45.0	44.8	33.1	41.1	45.2	40.8	44.6	49.7	45.5	47.8	44.9	43.4	44.1
	C12	26.2	41.2	49.4	41.1	42.5	49.9	44.3	43.5	46.4	55.6	44.3	53.2	45.3	38.9	49.5	47.8	47.5	49.5	45.9	47.7
	C13	35.3	44.3	50.8	50.7	47.6	56.2	54.5	57.0	53.2	59.6	57.9	56.2	47.9	50.3	55.4	55.7	56.6	46.3	46.4	46.3
	C14	35.9	42.1	50.8	43.8	48.8	56.2	52.9	54.7	60.1	73.8	68.3	67.7	49.4	43.8	52.9	53.6	58.8	52.3	44.1	48.2
e fed, 24weeks		*n=18*	*n=18*	*n=18*	*n=18*	*n=18*	*n=18*	*n=17*	*n=17*	*n=17*	*n=17*	*n=17*	*n=17*	*n=17*	*n=17*	*n=17*	*n=17*	*n=17*	*n=17*	*n=17*	*n=17*
	FF1	38.2	46.2	57.7	52.8	50.0	58.2	44.2	39.8	55.5	56.5	43.8	47.8	38.5	45.5	55.3	47.2	50.8	59.9	48.9	48.0
	FF2	36.2	36.0	49.4	44.0	39.1	43.1	38.8	42.9	39.9	41.2	32.6	43.3	35.4	39.9	33.8	33.5	43.2	46.3	44.2	40.1
	FF3	40.3	32.9	47.6	46.4	40.6	48.7	41.2	41.2	48.6	41.7	31.3	42.0	37.1	33.1	47.1	35.7	42.2	48.0	42.5	40.5
	FF4	33.4	32.6	44.6	38.6	33.2	38.5	35.0	37.2	41.0	36.5	28.7	35.8	27.8	30.6	31.7	32.1	42.0	43.3	41.3	39.6
	FF5	38.8	49.2	50.4	46.2	51.3	64.4	48.8	61.4	60.5	47.5	44.0	56.1	43.4	51.3	56.1	53.6	56.5	68.2	54.6	58.8
	FF6	35.3	38.9	46.7	44.2	46.2	50.4	41.1	48.1	49.4	44.6	38.1	52.9	42.2	52.8	51.1	51.1	55.5	60.4	52.8	52.9
	FF7	37.1	44.6	53.8	41.8	48.6	52.1	50.9	55.4	58.7	45.5	33.0	50.0	24.9	20.5	45.6	46.8	49.0	58.1	46.1	45.6
	FF8	35.1	41.0	49.6	48.5	55.4	66.4	55.0	56.9	68.6	51.3	44.2	60.6	46.3	31.4	27.4	54.4	61.9	73.0	66.8	69.1
	FF9	26.1	33.4	40.7	39.4	31.8	41.7	31.0	33.6	30.5	32.7	29.3	57.0	35.8	28.8	25.3	30.5	31.8	41.9	36.6	29.6
	FF10	39.7	57.7	58.8	55.9	45.8	47.9	34.8	40.2	43.8	51.1	35.5	35.3	38.2	38.6	43.5	42.1	45.6	56.6	50.5	49.8
	FF11	33.4	36.5	41.6	44.6	45.0	53.7	41.2	54.1	50.0	43.1	47.1	55.0	33.0	44.0	53.6	44.9	68.9	63.5	45.3	58.5
	FF12	33.3	41.7	44.4	45.2	40.3	41.1	41.8	44.5	48.7	41.5	38.3	47.2	36.4	36.3	39.0	42.5	43.5	44.9	32.6	33.2
	FF13	28.6	35.4	49.4	45.0	32.4	43.0	34.2	36.7	38.1	34.7	29.1	40.9	31.1	30.5	25.9	19.2	30.1	51.7	37.2	45.1
	FF14	32.1	37.2	40.7	38.3	30.8	42.5	52.1	56.1	62.2	55.7	38.7	54.7	30.9	44.5	47.5	52.1	56.7	62.7	49.9	49.9
	FF15	23.0	37.9	50.4	40.0	51.1	62.6	56.6	65.7	64.5	58.4	39.2	68.4	42.8	48.0	50.9	59.9	55.5	61.4	55.0	54.0
	FF16	33.6	48.9	64.1	52.5	59.3	69.1	57.0	64.3	70.8	71.7	42.5	77.0	52.0	55.0	46.6	50.0	51.9	65.3	80.5	77.5
	FF17	39.6	51.8	59.9	59.2	59.6	70.7	35.9	42.2	46.1	33.6	33.7	31.8	36.0	36.5	39.2	35.4	38.4	50.1	50.0	51.0
	FF18	33.1	43.5	42.3	40.3	38.0	47.9	D	D	D	D	D	D	D	D	D	D	D	D	D	D
