# Supplementary figures and images for: Metabolic Syndrome and Hypertension Resulting from Fructose Enriched Diet in Wistar Rats
Source: Biomed Res Int. 2017 Apr 11;2017:2494067. doi: 10.1155/2017/2494067 (PMC5405603; doi:10.1155/2017/2494067)

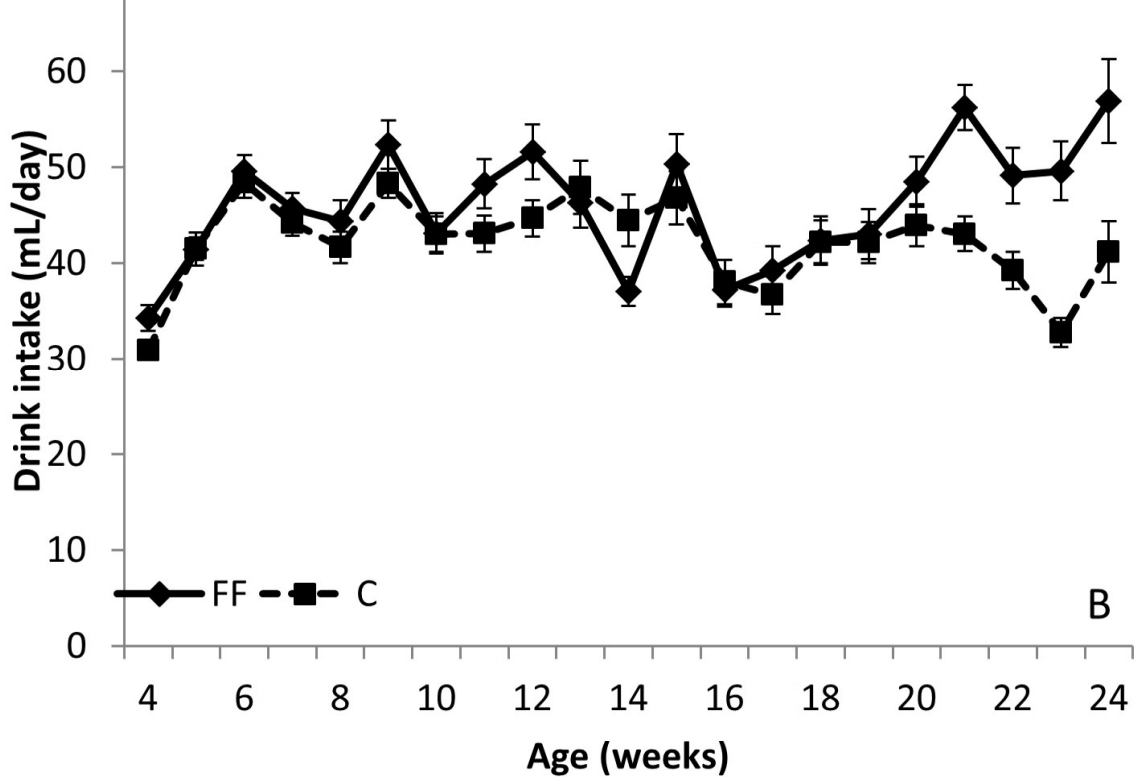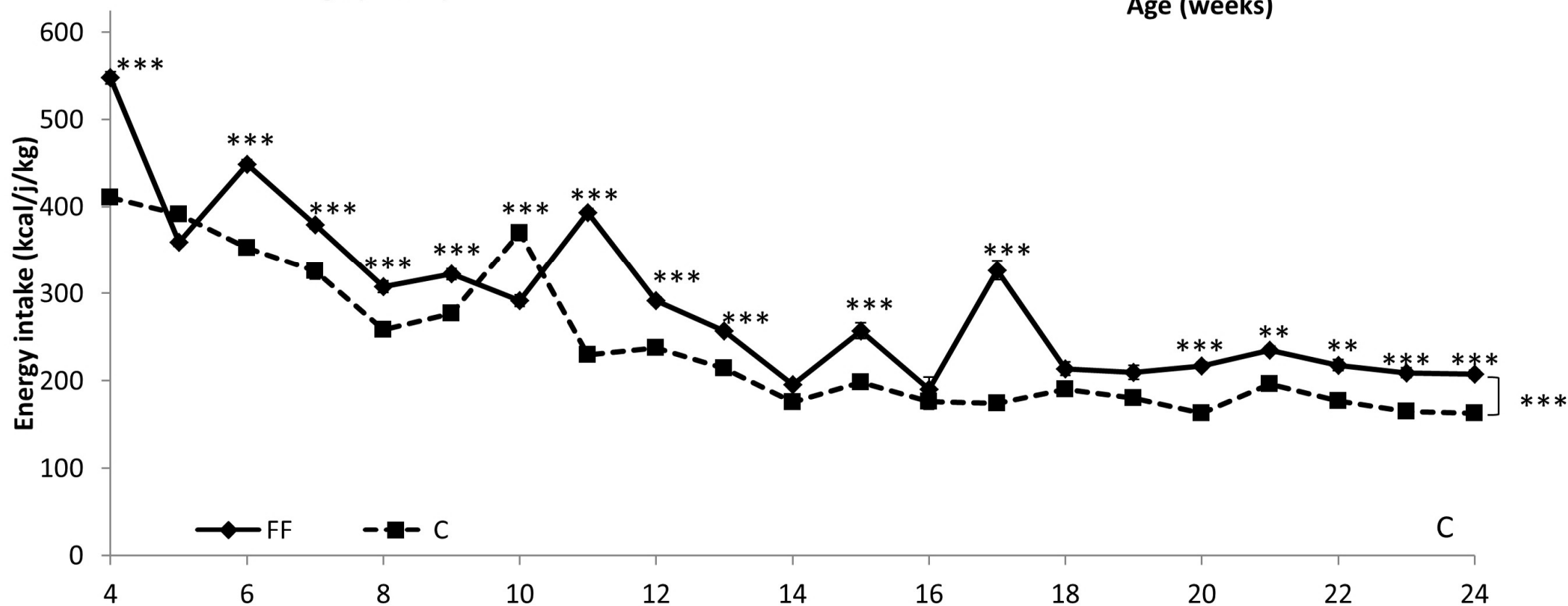

Supplement: Supplementary file 1 — S1 Fig. Effects of fructose consumption on food (A), drink (B) and energy intake (C). Energy intake (kcal/day/kg of bodyweight) was calculated from the food (kcal/day) and drink (mL/day) intake. S1 Table. Raw data for drink intake (mL/day). S2 Table Raw data for food intake (g/day). [file 2494067.f1.zip › Supplementary files pdf/Figure S1.pdf]
