# Supplementary material for: Metabolic Syndrome and Hypertension Resulting from Fructose Enriched Diet in Wistar Rats
Source: Biomed Res Int. 2017 Apr 11;2017:2494067. doi: 10.1155/2017/2494067 (PMC5405603; doi:10.1155/2017/2494067)
Supplement: Supplementary file 1 — S1 Fig. Effects of fructose consumption on food (A), drink (B) and energy intake (C). Energy intake (kcal/day/kg of bodyweight) was calculated from the food (kcal/day) and drink (mL/day) intake. S1 Table. Raw data for drink intake (mL/day). S2 Table Raw data for food intake (g/day). [file Table 1.pdf › Supplementary files pdf/Table 1.pdf]

		Food intake (gram/day)																				
	**Age (weeks)**	4	5	6	7	8	9	10	11	12	13	14	15	16	17	18	19	20	21	22	23	24
**Group**	**Rat**																					
Control, 24weeks		*n=14*	*n=14*	*n=14*	*n=14*	*n=14*	*n=14*	*n=14*	*n=14*	*n=14*	*n=14*	*n=14*	*n=14*	*n=14*	*n=14*	*n=14*	*n=14*	*n=14*	*n=14*	*n=14*	*n=14*	*n=14*
C	C1	17.7	22.7	26.6	29.1	25.5	30.6	30.0	30.3	31.1	28.0	21.4	26.1	27.3	28.3	30.8	30.5	27.7	33.9	31.3	30.0	30.7
C	C2	17.5	23.7	27.9	29.0	27.7	34.1	30.3	28.9	32.1	30.8	25.6	20.2	19.2	22.5	28.8	29.5	27.5	33.3	31.6	26.7	29.1
C	C3	17.4	18.2	21.6	24.1	21.5	26.5	24.4	21.4	24.6	23.6	19.9	25.3	21.6	18.7	23.6	21.7	20.6	25.4	24.7	21.3	23.0
C	C4	19.5	26.0	26.9	28.6	26.6	30.5	26.9	28.5	31.0	28.8	24.2	29.7	24.5	21.5	28.4	27.1	24.3	30.3	28.3	29.5	28.9
C	C5	18.3	25.1	29.0	32.7	30.5	35.5	31.1	31.7	35.6	37.2	31.7	34.1	32.5	31.4	31.6	25.3	28.0	36.7	35.0	34.8	34.9
C	C6	18.1	22.3	23.4	22.6	21.9	25.2	23.6	25.1	26.9	24.9	22.0	22.5	10.7	18.0	24.7	25.8	24.4	28.9	26.9	25.3	26.1
C	C7	19.0	26.6	30.5	30.0	28.3	34.8	30.9	33.0	34.9	33.6	29.4	36.0	28.1	27.6	29.8	26.3	27.9	35.2	33.0	30.8	31.9
C	C8	18.9	24.2	25.8	26.8	24.1	29.8	23.5	26.7	30.2	28.6	20.5	25.2	24.5	25.3	27.7	26.6	23.2	30.8	28.2	23.1	25.7
C	C9	20.2	27.7	31.9	33.2	31.1	37.9	31.0	32.0	37.1	31.0	26.9	35.7	32.7	32.1	31.4	32.3	30.6	37.9	20.4	27.0	23.7
C	C10	18.1	23.6	27.6	28.1	24.8	30.3	27.3	26.4	30.8	30.2	27.7	32.5	27.2	28.7	33.4	31.1	28.1	32.0	31.8	28.1	29.9
C	C11	19.9	25.7	26.8	26.1	25.6	31.4	27.0	29.2	32.0	18.0	16.5	27.3	28.4	27.4	32.3	30.4	26.7	30.7	29.1	28.9	29.0
C	C12	18.1	23.5	27.2	27.5	26.1	33.1	30.1	28.9	33.6	34.6	29.1	34.3	30.5	29.1	35.0	32.9	29.0	35.0	33.5	31.0	32.3
C	C13	17.5	24.2	27.6	29.4	30.4	36.4	32.4	33.0	35.4	32.8	28.1	33.9	28.3	29.3	32.7	34.3	30.4	34.6	31.2	33.4	32.3
C	C14	17.2	21.4	26.7	28.3	28.5	33.3	31.2	31.7	37.3	35.6	32.2	35.1	30.4	30.0	34.0	35.3	32.1	42.5	36.7	34.3	35.5
Fructose fed, 24weeks		*n=18*	*n=18*	*n=18*	*n=18*	*n=18*	*n=18*	*n=17*	*n=17*	*n=17*	*n=17*	*n=17*	*n=17*	*n=17*	*n=17*	*n=17*	*n=17*	*n=17*	*n=17*	*n=17*	*n=17*	*n=17*
FF	FF1	19.8	23.9	25.9	22.3	23.3	26.2	23.8	23.3	27.9	26.1	17.6	21.4	16.9	19.2	23.2	23.4	22.0	23.3	23.3	21.0	23.5
FF	FF2	17.6	21.4	23.4	24.0	21.6	23.4	21.1	20.5	22.6	20.8	14.9	20.1	17.9	19.0	19.2	18.2	20.6	19.9	20.0	19.2	21.3
FF	FF3	17.4	20.8	21.3	21.6	20.2	22.1	20.3	18.0	22.2	19.3	15.0	18.5	16.9	17.7	20.9	17.0	19.3	19.4	19.8	18.9	19.0
FF	FF4	15.1	19.0	21.2	20.9	19.3	20.7	19.9	19.1	21.5	20.0	14.8	19.8	13.4	16.6	18.4	17.5	18.5	18.3	18.3	18.0	18.1
FF	FF5	16.0	20.1	22.8	20.1	19.3	22.8	19.7	18.4	23.1	19.6	14.9	20.2	16.6	18.4	20.1	18.6	18.5	19.3	19.6	18.4	19.4
FF	FF6	13.9	19.9	20.8	22.1	18.6	20.2	18.9	18.2	21.2	18.1	13.2	20.8	18.3	19.5	20.5	18.2	16.2	15.3	18.3	15.6	15.7
FF	FF7	17.5	21.1	25.3	24.6	23.2	27.1	25.5	22.6	26.3	23.5	17.4	23.7	13.1	18.4	20.9	23.2	24.3	24.3	21.7	21.2	21.2
FF	FF8	14.8	22.0	21.3	19.3	19.5	19.9	18.2	17.0	19.1	18.0	13.8	18.4	13.8	16.1	8.7	11.2	14.5	16.3	17.7	15.9	15.0
FF	FF9	15.1	19.1	23.4	22.9	19.3	23.9	20.2	17.2	22.5	22.1	14.5	20.1	20.5	20.3	16.4	18.4	15.9	20.5	21.8	17.4	19.4
FF	FF10	16.5	19.7	24.2	22.8	19.8	22.8	20.9	20.5	22.0	23.1	18.1	18.9	17.9	18.4	18.3	17.4	17.1	17.9	17.1	13.9	18.3
FF	FF11	17.7	21.8	22.3	21.3	19.8	23.3	20.5	19.9	21.6	20.0	16.9	21.2	16.0	18.6	19.8	18.8	19.4	19.1	19.8	17.7	18.0
FF	FF12	17.6	21.2	23.8	24.4	21.4	24.5	19.3	18.6	22.3	21.3	15.7	21.4	21.4	21.4	22.0	19.5	18.6	21.2	18.5	18.0	21.1
FF	FF13	17.3	22.1	22.6	20.8	20.8	25.3	22.9	21.3	25.8	22.3	16.4	24.1	21.6	22.8	21.6	9.3	16.3	22.0	20.4	20.4	21.0
FF	FF14	15.7	20.4	23.6	22.3	23.2	26.4	20.8	18.4	21.7	19.5	14.3	19.6	15.0	17.3	16.9	16.2	16.1	17.6	16.2	16.0	16.8
FF	FF15	15.2	20.7	24.4	22.9	20.6	26.2	18.2	17.1	21.1	17.1	16.7	20.5	15.7	18.1	20.3	19.7	18.5	17.6	17.8	15.7	15.8
FF	FF16	17.0	22.3	25.0	22.9	19.6	23.8	17.1	19.0	20.6	18.0	14.8	20.4	17.4	18.9	14.1	14.6	16.4	21.3	23.5	22.2	20.0
FF	FF17	16.9	22.3	24.3	22.8	21.3	23.0	21.4	21.0	24.1	26.8	16.0	21.4	19.7	20.5	24.1	22.9	22.3	21.6	21.6	21.5	21.6
FF	FF18	17.3	23.3	22.8	21.9	22.2	25.7	D	D	D	D	D	D	D	D	D	D	D	D	D	D	D
